# Anaesthetic management and complications of a Humboldt penguin (*Spheniscus humboldti)* undergoing diagnostic imaging

**DOI:** 10.1186/s13620-023-00256-7

**Published:** 2023-10-04

**Authors:** Patricia Romero, Flavia Restitutti, Niamh McGill, Seamus Hoey, Rachel C. Bennett

**Affiliations:** 1 University College, Dublin School of Veterinary Medicine, Belfield, Dublin 4, Ireland; 2 Dublin Zoo. Saint James’ (Part of Phoenix Park), Dublin 8, Ireland

**Keywords:** Anaesthesia, Diagnostic imaging, Humboldt penguin, *Spheniscus humboldti*, Tracheal bifurcation, Septum

## Abstract

**Background:**

The presence of a tracheal septum dividing the trachea into two makes intubation one of the main challenges of penguin anaesthesia. Differences in the length and location of the aforementioned tracheal septum have been described in some penguin species. However, to the best of the authors’ knowledge, it has not been reported in Humboldt penguins (*Spheniscus humboldti)*. Therefore, one of the aims of this publication is to report the septal position in this Humboldt penguin. Furthermore, this publication describes the anaesthetic protocol and complications encountered and discusses some of the more important features of penguin anaesthesia. It is anticipated that this case report will aid in future procedures requiring anaesthesia of this penguin species.

**Case presentation:**

A 25-year-old female Humboldt penguin was anaesthetized at the University College Dublin Veterinary Hospital for radiographs and computed tomography (CT) following three weeks of inappetence. After assessing the health status of the penguin from the clinical history and performing a physical examination, an American Society of Anesthesiologists physical status score of II was assigned and a combination of butorphanol 1 mg/kg and midazolam 1 mg/kg was administered intramuscularly to sedate the penguin. Induction of anaesthesia was performed via a face mask using sevoflurane in oxygen. The airway was intubated with a 4.0 mm Cole tube and anaesthesia was maintained with sevoflurane in oxygen during the entire procedure. Anaesthetic monitoring consisted of an electrocardiogram, pulse oximetry, non-invasive blood pressure, capnography, and body temperature.

**Conclusions:**

Tracheal bifurcation was identified as the start of the tracheal septum 4.67 cm from the glottis using CT. Most of the anticipated complications of penguin anaesthesia, such as hyperthermia, hypothermia, regurgitation, hypoventilation, and difficulties in intubation were present in this case. However, no major sequalae occurred following the anaesthetic protocol described.

## Background

There are a total of 18 different penguin species worldwide and the Humboldt penguin (*Spheniscus humboldti*) is one of the four species in the genus Spheniscidae: their closest relatives being the African (*Spheniscus demersus)*, Magellanic (*Spheniscus magellanicus)* and Galapagos penguins (*Spheniscus mendiculus*). Humboldt penguins reside in South America and inhabit the coastal regions of Peru and Chile, which are warm and temperate [1]. Humboldt penguins are described as medium-sized birds, approximately 56–70 centimetres (cms) in length/height with an approximate body weight of 4.2 kilograms (kg) [2]. The lifespan of Humboldt penguins is reported to be 15–20 years in the wild [3], although some individuals in captivity can live for up to 25–30 years [4]. Their conservation status is defined as vulnerable by the International Union for Conservation of Nature (IUCN) Red List of Threatened Species, since populations are decreasing in the wild [5]. Therefore, veterinary care of colonies in captivity is important. In addition to prophylactic care such as physical examination, blood collection and vaccination under manual restraint, sedation or general anaesthesia may be required for advance medical procedures such as diagnostic imaging and surgery.

Penguin species have anatomical and physiological adaptations, which enable them to forage and breed in some of the extreme conditions they inhabit. However, differences also exist between individual penguin species some of which are poorly documented in the literature making the anaesthetic management of specific penguin species challenging [3, 4, 6].

The anaesthetic considerations and anticipated problems associated with the anaesthesia of penguins include difficulties associated with intubation, regurgitation and aspiration, hyperthermia, hypoventilation and pre-existing conditions such as pulmonary aspergillosis. To the authors´ knowledge, only a few anaesthetic protocols have been reported thus far to sedate or anaesthetize Humboldt penguins. Induction with facemask and maintenance with isoflurane in oxygen, without previous sedation, was reported for anaesthesia of an 18-month-old Humboldt penguin prior to endoscopic foreign body removal [7]. Two other reports described the use of injectable agents to sedate Humboldt penguins: one report, based on ketamine at 5 mg/kg administered intramuscularly (IM) was found to be an effective sedative [8], whereas another report, showed that a combination of medetomidine 0.05 mg/kg, ketamine 5 mg/kg and butorphanol 0.5 mg/kg IM was effective to restrain Humboldt penguins undergoing non-invasive and minor painful procedures [9].

Some penguin species have a “double trachea” [10] formed by a septum dividing the trachea in two [4]. This bifurcation may be present or absent and can be located at different levels depending on the species [11]. In the *Spheniscus* genus the length is reported to be very variable [12] and the authors were unable to find information about the length and location of the septum in the Humboldt penguin.

This case report has two aims: the first, to describe the anaesthetic management of a geriatric Humboldt penguin for diagnostic imaging procedures, detailing the anaesthetic protocol used, the complications that occurred and their subsequent management. Moreover, the authors describe the upper airway features visualised with computed tomography (CT) and radiography. It was hoped that, after diagnostic imaging procedures, the dimension of the septum dividing the trachea and other measurements such as the tracheal internal diameter (ID) could be determined. Moreover, this information could prove helpful to veterinarians who need to anaesthetize Humboldt penguins in the future.

## Case presentation

A 25-year-old female Humboldt penguin presented to the Veterinary Hospital of the University College Dublin (UCDVH) for investigation of inappetence with a 3-week duration. The penguin had not produced any eggs for several years, which was attributed to the age.

On arrival at the UCDVH the physical examination findings of the penguin were as follows: demeanour was bright, alert, and responsive, heart rate (HR) was 140 beats/minute (bpm) based on cardiac auscultation, respiratory rate (RR) 12 breaths/minute by external visualization when in the carrier, mucous membranes – pink and body condition score – 3/5, based on a body condition scale for Magellanic Penguins [13]. American Society of Anesthesiologists (ASA) physical status score was classified as II and body weight was 3.9 kg.

Haematology performed by the referral veterinarian at the zoo showed no abnormalities and serum biochemical analyses showed hypercalcemia of > 4 mmol/L, equivalent to 16.03 mg/dL (reference range: 8.7–12.8 mg/dL) and mild disturbances of liver values: globulins 5.4 g/dL (0.6–5.3 g/dL), total proteins 7.4 g/dL (3.7–6.9 g/dL), ALT 231 U/L (11–105 U/L), total bilirubin 32 µmol/L, equivalent to 1.87 mg/dL (0–1.6 mg/dL) [3].

Ventrodorsal (VD) radiographs were taken at the referral centre 3 weeks before presentation with the penguin conscious, showing a well-defined mineral opaque round-lobulated structure superimposed on the region of the cloaca. Since this mineralisation may have indicated a retained egg due to uterine inertia, the penguin was treated with 300 mg of calcium (as calcium carbonate) and 5 µg of cholecalciferol (Caltrate, GlaxoSmithKline Consumer Healthcare Limited, Citywest Business Campus, Dublin 24, Ireland) orally once daily for the 2 weeks prior to presentation. Other medication consisted of: 500 mg co-amoxiclav (Kesium, Ceva Sante Animale, France) orally twice daily for 26 days, 1.75 mg meloxicam (Metacam, Boehringer Ingelheim, Vetmedica GmbH, 55,216 Ingelheim, Rhein, Germany) orally once daily for 22 days and 100 mg itraconazole (Sporanox, McGregor Cory Limited, Middleton Close, Banbury, Oxfordshire, UK) orally once daily for 12 days (to treat potential aspergillosis resulting from stress). Serum biochemistry tests were repeated 3 days after the initial sample, and globulins remained persistently elevated at 5.8 g/dL (0.6–5.3 g/dL), while ALT had decreased to 153 U/L (11–105 U/L) and total bilirubin remained persistently elevated at 48 µmol/L, equivalent to 2.81 mg/dL (0–1.6 mg/dL) [3].

The penguin had maintained her body weight over the course of the illness because of daily assisted feeding by the keepers but continued to be otherwise inappetent and lethargic despite the medical intervention.

The penguin presented for full body imaging—radiographs and CT—under general anaesthesia. Premedication consisted of butorphanol (Butador®; Chanelle Pharma, Loughrea, Galway, Ireland) 0.5 mg/kg combined with midazolam (Midazolam: Hypnovel®, Roche Products Ltd., Welwyn Garden City, UK) 1 mg/kg injected IM into the pectoral muscles. After 10 min, the degree of sedation was slight, and an additional 0.5 mg/kg of butorphanol was administered IM. Following 6 min the degree of sedation was moderate, and anaesthesia was induced with sevoflurane (Sevoflurane: Sevoflo®, Abbott Animal Health, Dublin, Ireland) -vaporizer setting 4–5% in oxygen delivered by a zero dead space face mask. The trachea was intubated with a Cole tube 3.5 mm ID (MILA Inter- national, INC; UK) and anaesthesia was maintained with a sevoflurane—vaporizer setting between 2 and 4.5%. Oxygen was supplied at 2.5 to 3 L/minute using a non-rebreathing circuit [Mapleson D (T-piece)], and manually assisted ventilation was started because the penguin experienced hypoventilation (Pe’CO_2_ 7 kPa). After 10 min of anaesthesia the penguin regurgitated a small quantity of gastric contents, which was suctioned, and the airway cleaned. The endotracheal tube (ETT) was leaking audibly when ventilation was assisted and it was decided to extubate and re-intubate with a larger tube, therefore the ETT was replaced with a 4 mm ID Cole tube (MILA Inter- national, Inc; UK).

Manual ventilation was continued with a RR between 4–15 breaths/minute for the entire procedure; however, a small audible leak from the ETT was still detected.

Anaesthesia monitoring consisted of oxygen haemoglobin saturation (SpO_2_) measured with a portable pulse oximeter probe attached to the flipper (LifeVet PT, Eickemeyer, Germany), end-tidal carbon dioxide partial pressure (Pe’CO_2_) via a side stream capnograph (Mindray BeneView T5 multiparametric monitor, India), HR was measured with both the pulse oximeter and an electrocardiogram (ECG) and non-invasive blood pressure (NIBP) was measured with an oscillometer (Suntech Vet20 Veterinary Blood Pressure Monitor, USA) with a number 2 cuff placed around the tarsus. Cloacal temperature was measured with a digital thermometer (TRO-DIGITERM, TROGE, Hamburg, Germany). Monitored values and reference ranges are summarised in Table 1.

**Table 1:** Physiological variables recorded during general anaesthesia of a 25-year-old female Humboldt penguin anaesthetized at UCDVH. Data are shown as minimum and maximum values

Variable	SpO_2_ (%)	PE’CO_2_ (mmHg) [kPa]	HR (bpm)	SAP (mmHg)	DAP (mmHg)	MAP (mmHg)	Temp (ºC)
Value (range)	96–100	25–56 [3.5–7.5]	120–200	100–160	46–89	65–98	35.5–39.6
Reference range	N/A	30–45 [4-6] [14]	121 ± 5 (resting) 139 ± 5(floating in water) 245 ± 24 (running) [15]	N/A	N/A	N/A	37.8–38.9 °C [3]

Full-body radiographs VD and lateral were taken. Inspection of the radiographs showed that the ETT was positioned within the left tracheal bifurcation. Therefore, the ETT was withdrawn slightly, and a second radiograph of the neck confirmed the correct placement of the ETT tube proximal to the bifurcation. A 24-gauge intravenous (IV) catheter (Intraflon 2, VYGON, Ecouen, France) was inserted into the right metatarsal vein.

**Fig. 1:**
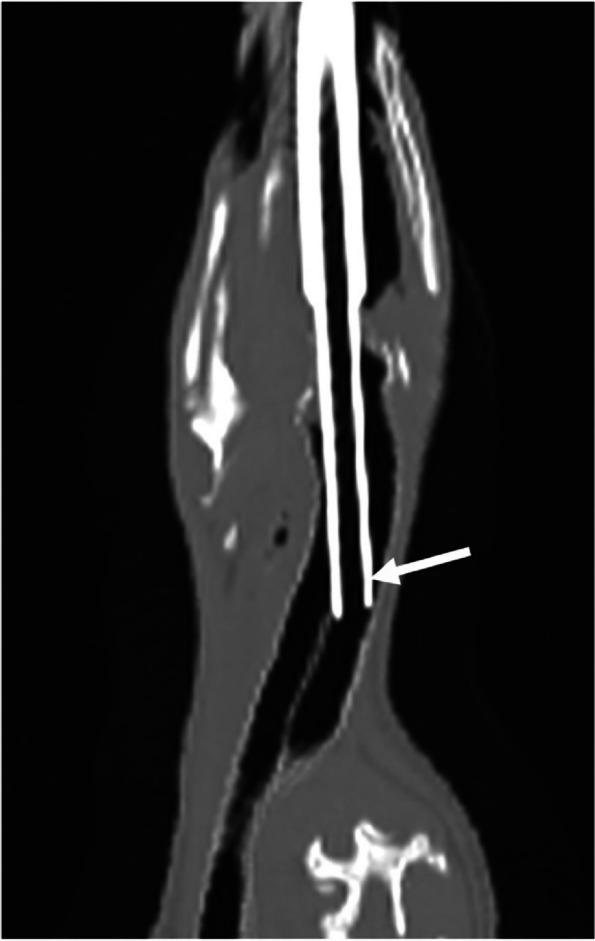
Dorsal oblique plane CT image showing the endotracheal tube (arrow) at the left tracheal bifurcation

Following the radiographs, the penguin was moved to perform a full body CT examination. Ioversol, an iodinated non-ionic contrast (Optiray 300® ioversol 300 mg I/mL, Guerbet, France) was administered via the IV catheter. CT images showed extravasation of contrast consistent with IV catheter dislodgement. Therefore, a second 24-gauge catheter was placed in the left metatarsal vein and a total of 4 mL of contrast (307 mg I/kg) were administered IV. CT images showed that the ETT was positioned slightly beyond the tracheal septal bifurcation located within the left trachea, despite being repositioned when radiographs were performed (Fig. 1).

**Fig. 2:**
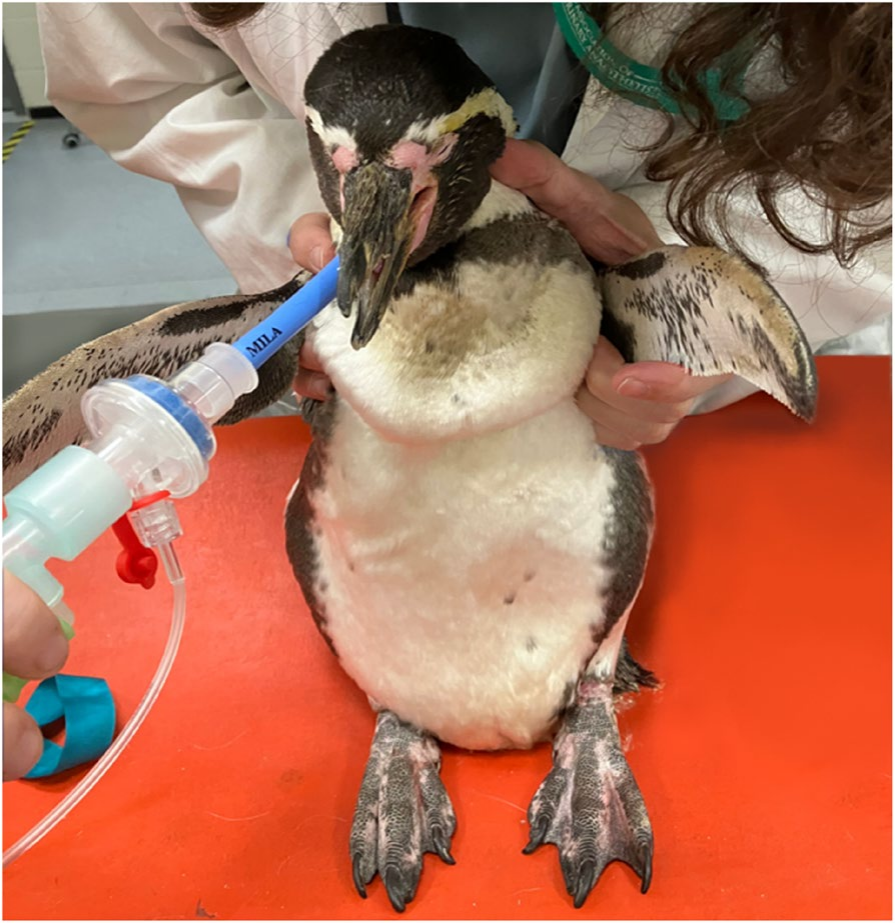
Penguin recovering in standing position to avoid regurgitation

After 125 min, sevoflurane administration was ceased, and flumazenil (Flumazenil, Fresenius, Kabi, Deuchland, GmbH, Germany) 0.04 mg/kg was administered IV. The penguin was maintained in a standing position for the recovery period (Fig. 2). Recovery was rapid and uneventful, despite mild ataxia during the first 15 min following extubation.

Based on the CT scan, a diagnosis of salpingitis was made and therefore antimicrobial treatment with co-amoxiclav 500 mg orally twice daily was continued. No other abnormalities were reported in the CT scan.

**Fig. 3:**
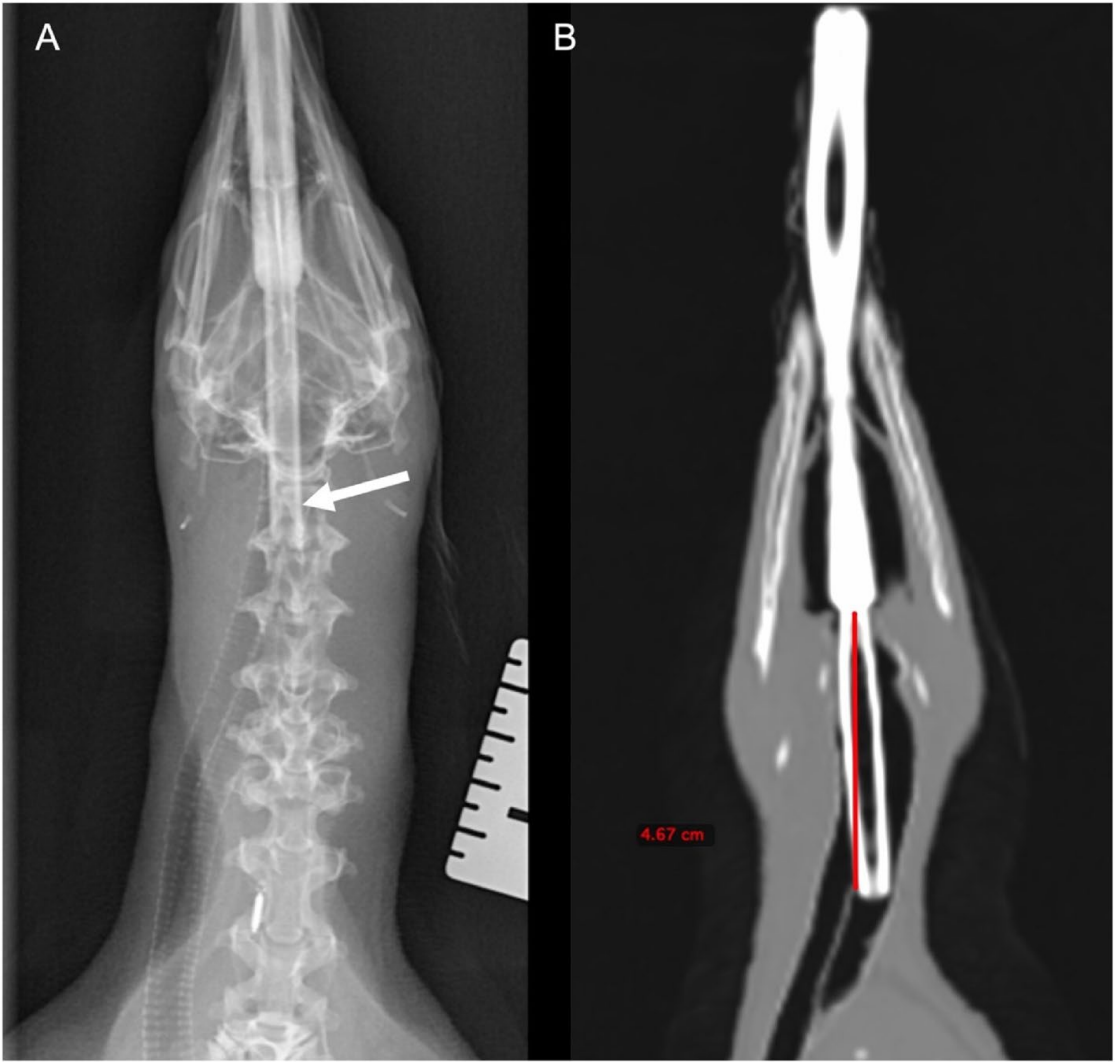
**A** Ventrodorsal radiograph identifying the endotracheal tube (arrow) at the level of the tracheal bifurcation. **B** Dorsal oblique plane CT image showing tracheal bifurcation at approximately 4.67 cm from the glottis

Radiographs of the penguin´s airway confirmed that this Humboldt penguin had a tracheal bifurcation, which began approximately 3.60 cm from the glottis (Fig. 3A). Radiographically, the glottis was superimposed by the calvarium preventing measurements. On CT images, the length from the glottis to the tracheal bifurcation was 4.67 cm (Fig. 3B).

**Fig. 4:**
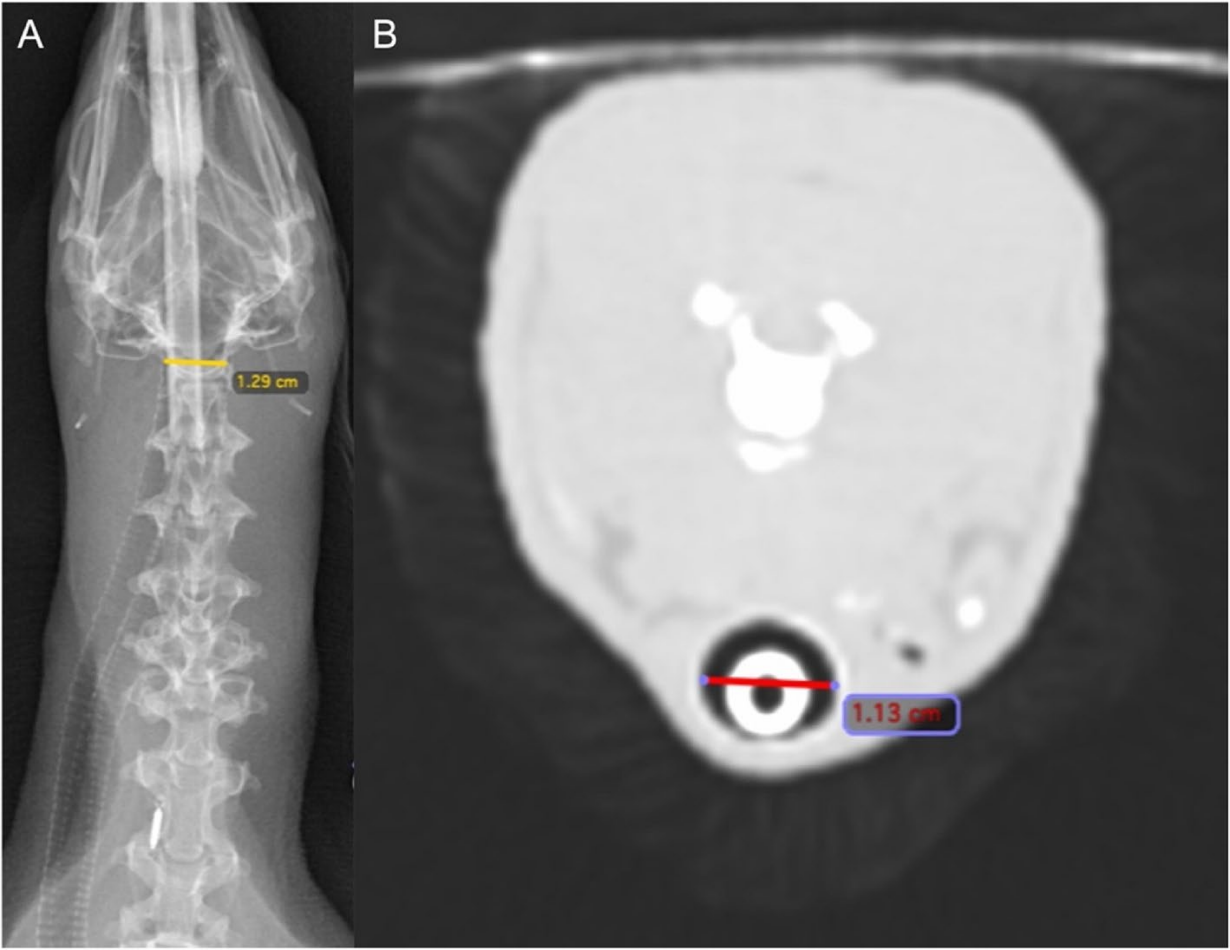
**A** Ventrodorsal radiograph of the head and neck showing the tracheal internal diameter (line). **B** Transverse CT image in lung window showing the proximal tracheal internal diameter (line)

Furthermore, the ID internal diameter of the proximal trachea was measured at approximately 1.29 cm on radiographs and 1.13 cm on CT (Fig. 4A and B).

## Discussions and conclusions

The Humboldt penguin described in this case report was 25 years old with a bodyweight of 3.9 kg therefore a small to medium sized geriatric penguin with a normal body weight. The ASA status was classified as II as the animal had mild systemic disease. Although a recent retrospective study in birds showed no difference in mortality outcomes when different ages were compared, the study showed that mortality rate increased when ASA status was equal to or greater than III, therefore suggesting that the associated anaesthetic risk is higher in less systemically well birds. So, in this case, the anaesthetic risk was low [16].

Handling stress owing to separation from the zoo (home) environment and members of the colony may worsen subclinical infections so sedation and anaesthesia may be required if a procedure needs to be performed.

### Pre-anaesthesia preparation

Fasting for a minimum of 8–12 h [6] and up to 18–24 h [4] is recommended to empty the stomach of penguins before anaesthesia [6] and thereby to prevent regurgitation and aspiration of gastric content as the risk is high. In addition, fasting reduces stomach and intestinal load and helps the bird to breath with less difficulty especially when in dorsal recumbency [3, 4, 17]. In this case, despite fasting for 19 h, the penguin regurgitated approximately 13 min after induction of anaesthesia, supporting the current literature. Therefore, a minimum of 24 h fasting, as described in previous literature [4], would be more appropriate to avoid this potential complication.

Pre-anaesthetic blood tests can help the anaesthetist to diagnose certain systemic diseases in penguins. Aspergillosis is a common fungal disease in birds with a clinical course that is mostly subacute or chronic [18]. It is triggered by stressful situations such as transportation away from the colony and anaesthetic procedures and is one of the most common causes of death in captive penguins [19, 20]. Haematologic findings such as leucocytosis, monocytosis, lymphopenia, or non-regenerative anaemia can be highly suggestive of aspergillosis [21]. However, in this case, haematology showed no abnormalities, decreasing the probability of this illness. In addition, this penguin was receiving itraconazole 20 mg/kg orally once daily as a prophylactic medication against *Aspergillus fumigatus.* Diagnostic imaging procedures such as radiography or CT are helpful tools to diagnose aspergillosis by identifying air sac granulomas or other changes in the lung fields [22, 23]. No evidence of aspergillosis was observed in the CT images of this individual. Serum biochemistry tests provide some information about renal and/or hepatic function. This is important because most of the commonly used anaesthetic and sedative agents are metabolized in the liver. In this case, moderate hypercalcemia, mild hyperproteinaemia, and a mildly increased ALT and total bilirubin were seen. Based on these results a combination of a short acting opioid, such as butorphanol and a reversible sedative agent, such as midazolam, were chosen as a balanced premedication before the administration of sevoflurane to maintain general anaesthesia.

### Premedication, induction and maintenance of anaesthesia—drugs

Although penguins tolerate minimal restraint quite well for minimally invasive procedures [4], some procedures (advanced imaging and surgical procedures) require sedation or general anaesthesia.

In this case, the penguin was initially premedicated with midazolam and butorphanol, to minimize stress during manipulation and induction of anaesthesia. Premedication also provides analgesia and allows reduction of inhalant anaesthetic requirements in wild and captive animals [24]. The use of opioids such as butorphanol (1 mg/kg IM) was an effective premedication before inhalant anaesthesia in an African penguin (*Spheniscus demersus*) undergoing hemivertebra surgical repair [25]. Butorphanol administered IM, at a dose of 0.5 mg/kg is also reported in Humboldt penguins, in combination with medetomidine 0.05 mg/kg and ketamine 5 mg/kg [9]. This protocol, however, was associated with a short period of muscle relaxation and limited tolerance to endotracheal intubation. In addition, a decrease in HR was also reported in the aforementioned study [9], which had to be avoided in this case due to the advanced age of the penguin. Instead, midazolam was administered with butorphanol due its cardiovascular stability, sedative effects in birds and the potential to prevent or reduce the stress response during induction of anaesthesia with halogenated volatile anaesthetic drugs [26]. The dose of 1 mg/kg was based on personal communication with colleagues. The initial sedation was deemed light 10 min after the IM administration, since the penguin was showing signs of stress inside the cage; an additional 0.5 mg/kg of butorphanol was administered IM. The total dose of 1 mg/kg of butorphanol agrees with a previous report in another species of the *Spheniscus* genus [25].

As several procedures were performed involving changes in position and location of the penguin (CT and radiography), the bird was intubated to protect the airway. In addition, intubation will allow the provision of a high inspired fraction of oxygen (FiO_2_) and will also permits the anaesthetist to assess Pe’CO_2_ with capnography.

The most commonly used protocols to induce and maintain anaesthesia in penguins involve the administration of inhalant anaesthetic agents [6]. Sevoflurane was used instead of isoflurane due its relatively low blood:gas partition coefficient and the faster induction and recovery times. In addition, it has been shown to maintain a higher RR in a study in birds [6, 27]. Moreover, due to the less pungent odour compared to isoflurane, sevoflurane may provide a smoother induction of anaesthesia [6].

When inhalant anaesthetics are used for induction, apnoea or shallow breathing and bradycardia can occur in diving birds as a cardiorespiratory diving response or dive reflex [4, 28]. In this case, the fact that midazolam and butorphanol were used as sedative agents before induction, could be the reason why this penguin did not experience any bradycardia, or apnoea or other complications secondary to a stress response during induction.

### Intubation and airway management

As mentioned above, some species of penguins have a septum that divides the trachea cranially from the bronchial bifurcation into right and left, often called a “double trachea” [10, 29]. In the *Spheniscus* genus, which includes the Humboldt penguin, the length from the glottis to the start of the tracheal septum is reportedly very variable [11, 12]. The authors were unable to find the specific length of the tracheal septum in this species prior the anaesthetic event. For the anaesthetist, this will add an extra challenge since the presence of the bifurcation can lead to unilateral tracheal intubation with the risk of trauma to the tracheal septum if an inappropriate endotracheal tube size or length is chosen [4, 10]. In addition, it is worth mentioning that unilateral tracheal intubation does not pose a significant risk to the penguin’s ventilation as avian species have an efficient pulmonary-air sac system to achieve gas exchange [26, 30]. For this reason, and compared to other species such as mammals, unilateral ventilation does not lead to hypoxaemia even if the penguin hypoventilates.

In the present case, the measured distance from the glottis to the tracheal septum was approximately 3.60 cm when radiographs were used, whereas when the measurement was performed using the CT images, the distance was 4.67 cm. This discrepancy of almost 1 cm is due to the glottis being superimposed by the skull in the radiographs and is an important finding since it indicates that the ETT could still advance beyond the septum if radiographs alone were taken and used. It is important to note that the measurements performed in this case report cannot be extrapolated to other penguin species.

Previous literature reports that while in the Rockhopper penguin (*Eudyptes chrysocome*) the septum projects only 5 mm in length from the carina [12], in the Jackass penguin (*Spheniscus demersus)* the septum starts from only 1 to a few centimetres from the larynx [10, 11]. It has also been reported that in the Yellow-eyed penguin (*Megadyptes antipode*), the lower third of the trachea is also divided by a septum [20], and that in King penguins (*Aptenodytes patagonicus*), the septum extends over 80% of the tracheal length [31]. Therefore, these variations between species make these findings more relevant as it is the first time that the tracheal septum location has been measured in Humboldt penguins.

Similarly, to other bird species, the use of cuffed ETTs is not recommended in penguins as they can have partially ossified tracheal rings [31]. In addition, the use of Cole tubes has been reported as a safe method for endotracheal intubation when there is a risk of tracheal damage [32]. In this case, a first attempt with a 3.5 mm Cole endotracheal tube resulted in some air leakage during manual intermittent positive pressure ventilation (IPPV), so a 4 mm Cole endotracheal tube was replaced. This size of the ETT was comparable to a previous report in which a 4.5 kg Humboldt penguin was intubated with an uncuffed 4 mm ETT for endoscopic foreign body removal [7]. Despite this, a small leak was still present in this case when IPPV was performed so a slightly larger tube would have been a preferable option. Unfortunately, a larger size Cole tube was unavailable at the hospital. This presumption is supported by another report in which a smaller penguin: a 2.6 kg African Black-Footed penguin (*Spheniscus demersus*) was intubated with a 4 mm Cole endotracheal tube [33]. However, after measuring the ID of the trachea (see Fig. 4A and B) nothing larger than a 5 mm Cole tube [34] (with an outer diameter of 1.26 cm) would have been suitable for this penguin’s trachea.

### IV access

**Fig. 5:**
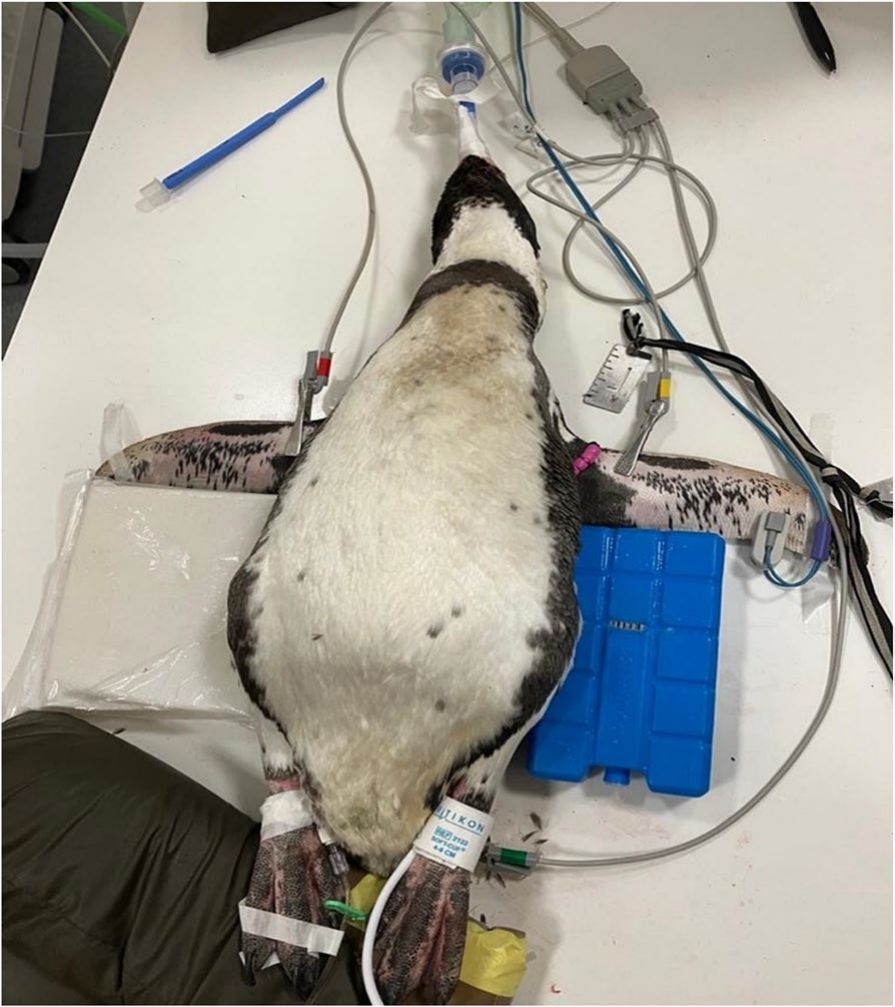
Humboldt penguin during general anaesthesia. Monitoring equipment, IV access and cold packs preventing hyperthermia

Catheterization for IV access can be performed in different locations such as the flipper vein (brachial or medial) [2, 35] or in the medial metatarsal vein [25] (Fig. 5). In the present case, both metatarsal veins were catheterized due to dislodgement of one catheter. Metatarsal vein catheters have been used in previous reports in penguins; [7, 25] however, there is a risk of faecal contamination due its proximity to the cloaca [6]. Despite this, no complications were experienced other than the dislodgement following the first catheter placement.

### Anaesthetic monitoring

Careful monitoring of anaesthesia is recommended when anaesthetizing penguins and specific considerations must be taken. Pulse oximetry has shown to be a deceptive method of measuring oxygen saturation in avian species owing to differences in the uptake features of oxygenated and deoxygenated haemoglobin. Despite this, pulse oximetry has some advantages when used to monitor avian patients, as it has a high accuracy in monitoring pulse rates up to 500 bpm with satisfactory recording of trends in oxygen saturation and HR [36].

The use of oscillometry to measure NIBP is unreliable when compared to direct blood pressure measurement in some bird species [37]. In the present case, NIBP was measured using a number 2 cuff placed around the tarsus (see Table 1 for measured values during anaesthesia). Although the measured values lack reference ranges for comparison, the pulse rate recorded by the oscillometer consistently matched the HRs given by the ECG and pulse oximeter readings throughout the procedure. Consequently, the oscillometer was employed to monitor trends and ensure the maintenance of an appropriate plane of anaesthesia during the procedure.

RRs and capnography values should be closely monitored to assess ventilation of anaesthetized patient. A previous study in anaesthetized African grey parrots using inhalant anaesthetics, indicated an adequate ventilation of the birds when Pe’CO_2_ values were between 30 to 45 mmHg [14]. Based on that study, ventilation was manually controlled to treat hypoventilation and ensure oxygenation of the patient.

Regarding HR monitoring, an earlier study described normal HRs in Humboldt penguins when they were resting, floating inactively in the water and when running, ranging from 121 to 245 bpm (Table 1). They also rated the frequencies under submersion (from 119–125 bpm) and after 60 s under the water (decreasing by 78 bpm) [15]. Based on the reported HRs, the penguin was maintained within normal limits during all the procedure, oscillating between 120 and 200 bpm (Table 1).

### Body temperature and thermoregulation

Normal body temperature in a penguin is reportedly between 37.8–38.9 °C [3]. They have been shown to develop both hypothermia and hyperthermia in the peri anaesthetic period [3, 4, 6, 38]. Core body temperature can be monitored in avian species via oesophageal or cloacal temperature probes. The oesophageal probe is inserted through the mouth and measures temperature at the level of the thoracic oesophagus [39]. If this probe is not available, or the procedure does not allow its use, alternatively a cloacal temperature probe can be used. However, cloacal temperature probes can be easily dislodged if they are not appropriately secured, with the consequence that measured temperatures appear to be lower [40]. But both measurement techniques can be correlated with one another and are non-invasive techniques [39]. In the present case, cloacal temperature was intermittently monitored via a digital thermometer since the presence of the ETT in the mouth impeded the use of an oesophageal probe.

The presence of the humeral arterial plexus in the flippers works as a heat exchanger system that limits heat loss through the flippers and enables the penguin to maintain and regulate their body temperature. In addition, the insulation provided by their feathers and the presence of a subcutaneous fat layer, also help them to maintain their body temperature [3, 6]. These protective mechanisms make them prone to hyperthermia during stressful conditions or during anaesthesia, and they can develop some cardiac arrhythmias and increase oxygen demand [26]. To reduce the risk of hyperthermia, some authors recommend the use of ice blocks (Fig. 5) on their feet and flippers [4, 6]. Another prophylactic alternative to prevent hyperthermia is to keep them in an air-conditioned environment to prevent fluctuations in temperatures [6]. In this case, the penguin experienced a period of hyperthermia (up to 39.6 ºC) at the beginning of the procedure that was treated by placing cold packs next to the flippers and the body of the patient.

However, there is also a risk of hypothermia during anaesthesia secondary to the inhalation of dry cold oxygen, [35] so constant temperature monitoring during anaesthesia is required. This patient experienced some periods of hypothermia (around 35.5 ºC) that were monitored and treated with the removal of the ice packs and passive warming techniques (blankets and towels) to decrease the heat loss.

### Positioning and recovery period

Some body positions of the penguin during anaesthesia negatively affect ventilatory function. A study in King penguins (*Aptenodytes patagonicus*) showed that they tend to develop tracheal obstruction because of the saliva accumulation while in dorsal recumbency, whereas when in ventral recumbency, there was less frequency of apnoeic episodes and “head lifting” to try to swallow accumulated saliva [38]. In this case, no complications were experienced related to the patient's positioning despite most of the procedure being performed in dorsal recumbency.

An animal’s position during recovery is also an important anaesthetic consideration. Some authors recommend a standing position to prevent regurgitation and aspiration pneumonia when the patient has not fully recovered all the laryngeal reflexes [6]. In this case, the maintenance of an upright position while still intubated was decided until the penguin started to move the head and extubation was possible (see Fig. 2). This position also prevented trauma of the flippers in case of excitation or excessive movement during the recovery period.

To hasten the recovery, flumazenil can be administered at the end of the procedure if benzodiazepines are given. This allows a faster recovery and the reversal of side effects if they are still present [4]. In this case, the penguin was extubated 6 min after the administration of flumazenil IV and recovery was considered uneventful and fast.

## Conclusions

Humboldt penguin anaesthesia can be a challenging procedure. Hyperthermia, hypothermia, regurgitation, hypoventilation, and difficult intubation are some of the anticipated complications of penguin anaesthesia and they were all present in this case. The anaesthetic protocol used, based on a premedication of butorphanol and midazolam IM and induction and maintenance with sevoflurane, showed no major complications and enabled smooth induction and recovery periods. Tracheal septum position in this Humboldt penguin is described, being approximately 3.60 cm from the glottis using radiographs and 4.67 cm from the glottis using CT images. Tracheal diameter was also measured, being 1.13 cm.

## Data Availability

The data used are available from the corresponding author on reasonable request.

## Abbreviations

CT: Computed tomography
IM: Intramuscularly
HR: Heart rate
Bpm: Beats per minute
RR: Respiratory rate
ID: Internal diameter
ETT: Endotracheal tube
SpO_2_: Oxygen haemoglobin saturation
Pe’CO_2_: End-tidal carbon dioxide partial pressure
ECG: Electrocardiogram
NIBP: Non-invasive blood pressure
IV: Intravenous
FiO_2_: Inspired fraction of oxygen
IPPV: Intermittent positive pressure ventilation
UCDVH: University College Dublin Veterinary Hospital
ASA: American Society of Anaesthesiologists
ALT: Alanine transaminase
